# Identification of ICF categories relevant for nursing in the situation of acute and early post-acute rehabilitation

**DOI:** 10.1186/1472-6955-7-3

**Published:** 2008-02-18

**Authors:** Martin Mueller, Christine Boldt, Eva Grill, Ralf Strobl, Gerold Stucki

**Affiliations:** 1 ICF Research Branch, WHO FIC Collaborating Center (DIMDI), Institute for Health and Rehabilitation Sciences, Ludwig-Maximilians-Universität, Munich, Germany; 2 Swiss Paraplegic Research, Guido A Zaech Institute, Nottwil, Switzerland; 3 Department of Physical Medicine and Rehabilitation, University Hospital Munich, Ludwig-Maximilians-Universität, Munich, Germany

## Abstract

**Background:**

The recovery of patients after an acute episode of illness or injury depends both on adequate medical treatment and on the early identification of needs for rehabilitation care. The process of early beginning rehabilitation requires efficient communication both between health professionals and the patient in order to effectively address all rehabilitation goals. The currently used nursing taxonomies, however, are not intended for interdisciplinary use and thus may not contribute to efficient rehabilitation management and an optimal patient outcome. The ICF might be the missing link in this communication process. The objective of this study was to identify the categories of the International Classification of Functioning, Disability and Health (ICF) categories relevant for nursing care in the situation of acute and early post-acute rehabilitation.

**Methods:**

First, in a consensus process, "Leistungserfassung in der Pflege" (LEP) nursing interventions relevant for the situation of acute and early post-acute rehabilitation were selected. Second, in an integrated two-step linking process, two nursing experts derived goals of LEP nursing interventions from their practical knowledge and selected corresponding ICF categories most relevant for patients in acute and post-acute rehabilitation (ICF Core Sets).

**Results:**

Eighty-seven percent of ICF Core Set categories could be linked to goals of at least one nursing intervention variable of LEP. The ICF categories most frequently linked with LEP nursing interventions were respiration functions, experience of self and time functions and focusing attention. Thirteen percent of ICF Core Set categories could not be linked with LEP nursing interventions. The LEP nursing interventions which were linked with the highest number of different ICF-categories of all were "therapeutic intervention", "patient-nurse communication/information giving" and "mobilising".

**Conclusion:**

The ICF Core Sets for the acute hospital and early post-acute rehabilitation facilities are highly relevant for rehabilitation nursing. Linking nursing interventions with ICF Core Set categories is a feasible way to analyse nursing. Using the ICF Core Sets to describe goals of nursing interventions both facilitates inter-professional communication and respects patient's needs. The ICF may thus be a useful framework to set nursing intervention goals.

## Background

The recovery of patients after an acute episode of illness or injury depends both on adequate medical treatment and on the early identification of needs for rehabilitation care. Acute rehabilitation is carried out by dedicated post-acute rehabilitation facilities, or by specialized wards within acute hospitals. Rehabilitation care in the acute situation is given individually by health professionals, mostly and typically by nurses with the goal to prevent complications and to restore functioning. In the early post-acute situation, rehabilitation is carried out by a multidisciplinary team, consisting of specialized health professionals, e.g. physiotherapists, occupational therapists, speech therapists, neuropsychologists, rehabilitation nurses and a rehabilitation physician. In early post-acute rehabilitation, in addition to their rehabilitation care, patients also have needs for ongoing medical and nursing care. The goal of acute and early post-acute rehabilitation is to prevent disability, to promote patients' autonomy and to avert the need for long-term care [1].

The rehabilitation process is a continuous and cyclic process in which health professionals are involved to comprehensively assess patients' functioning, assign patients to appropriate rehabilitation programs and interventions and to manage and evaluate these programs and interventions [2]. Despite of the interdisciplinary approach in rehabilitation, different professions use different, profession-specific taxonomies or classifications to describe relevant phenomena. Nursing professionals use, among others, the so called NNN-language system including the NANDA (North American Nursing Diagnosis Association) taxonomy to describe nursing diagnosis, the Nursing Interventions Classification (NIC) to describe nursing interventions and the Nursing Outcomes Classification (NOC) to describe nursing-related outcomes [3-5] or the International Classification of Nursing Practice (ICNP) [6] to describe diagnoses and interventions. As those systems were developed and used internationally, other approaches were developed by national or regional collaborations, e.g. the frequently used Swiss nursing workload classification "Leistungserfassung in der Pflege" (LEP) [7,8]. All these classification tools are useful in the context of communication and documentation among nurses, and well implemented in clinical practice. However, they are not intended for interdisciplinary use, and thus do not meet the necessity of efficient interdisciplinary teamwork in rehabilitation, where sharing gathered information on patients' functioning with all team members is substantive to efficient rehabilitation management and an optimal outcome [9]. A central point in managing the rehabilitation process is to define rehabilitation goals and to derive intervention targets based on a comprehensive assessment of patients' functioning [2].

Yet, many rehabilitation interventions are complex and have more than a single goal. To give an example, the nursing intervention of positioning a patient after stroke might have two goals: to prevent pressure sores and to stimulate correct muscle tone [10]. To date there is no general accepted standardized language in nursing to decompose complex goals of nursing interventions and to communicate them to other health professional groups in order to align them.

The International Classification of Functioning, Disability and Health (ICF) [11] is a multipurpose classification which belongs to the World Health Organization (WHO) family of international classifications and provides a comprehensive framework to draw a common picture of functioning, health and health-related domains. It was intended by the WHO to facilitate communication between different users such as health care workers, researchers, policy makers and the public.

However, there is evidence in the published literature that nursing professionals are not accustomed to the concepts of the ICF [12]. There are few studies reporting on the potential applicability of the ICF for nursing diagnoses [13,14], or describing goals of nursing interventions [15]. Kearney and Pryor (2004) outlined that the ICF is a potential framework for nursing that expands the dimensions of nursing thinking about health and disability [16]. Therefore, nursing classifications should be further investigated in respect of how they correspond to the ICF. Nursing interventions influence patients' functioning, and the ICF describes patients' functioning. Using the ICF to describe goals of nursing interventions might facilitate communication between all health professionals involved in the management of the rehabilitation process, and might enable goal-orientated collaboration.

The objective of this study was to identify the ICF categories relevant for nursing care in the situation of acute and early post-acute rehabilitation.

Specific aims were

(1) to identify ICF categories which can be linked with LEP nursing interventions.

(2) to identify LEP nursing interventions which can be linked with patients' functioning expressed by ICF categories.

## Methods

### Measures

#### LEP

LEP is a nursing workload classification for documenting the daily nursing activity. It is widely used in German-speaking countries because of the simple manageability in clinical practice and the corequisite standard language to document nursing resource utilisation in Switzerland [8]. The main part of this documentation tool is the "Nursing Interventions Catalogue", which contains 15 chapters of nursing areas (see Table 1). These chapters include a total of 79 different nursing interventions, which are detailed up to four specifications (e.g. very simple, basic, complex, very complex). Resultant, a total of 163 different nursing intervention variables are provided by the LEP [17]. They are structured by name, description, remarks, instructions and time allotment [7] (see Table 2 as an example). Goals concerning patients' functioning, which could be achieved when performing LEP nursing interventions, are not part of the LEP classification.

**Table 1:** LEP chapters of nursing areas

Movement	Personal Hygiene/Dressing	Eating/Drinking	Elimination
Respiration/Circulation	Documentation/Administration	Communications	Activity
Escort/Supervision	Safety	Hygiene	Conferencing
Specimen Management	Medication	Treatment	

**Table 2:** Example for LEP nursing intervention variable

**Mobilising straightforward**	**31.01**
**Description**	The patient receives straightforward support for mobilising
**Examples**	- straightforward mobilising after examination or minor surgery
	- mobilising at bedside
	- supervising prescribed mobilising
	- support for a straightforward transfer from chair or bed
	- mobilising with a tilt table
	- straightforward putting on of anti-thrombosis stockings
	- transfer to wheelchair
	- straightforward mobilising with crutches
	- straightforward holding of a baby or toddler
**Notes**	The variable includes all aspects of the mobilising process, including the use of any support aids which may be needed. It includes observing, accompanying and supporting the patient in order to promote independence/health.
**Instructions**	Note the distinction to the following variables: variables 54.17/18/19/20 (obtaining/fitting support aids)
**Comments**	-
**Time value**	5 minutes (multiple variable)

#### Acute and Post-acute ICF Core Sets

The ICF contains so-called ICF categories organized in two parts, each with two separate components. The first part covers functioning and disability with the components "Body Functions" (coded with b) and "Body Structures" (s), and "Activities and Participation" (d). The second part covers contextual factors with the components "Environmental Factors" (e) and "Personal Factors" [11]. The ICF categories of each component, with exception of the "Personal Factors", which are not classified yet, are hierarchically detailed up to four levels. The hierarchical code system consists of the abbreviation of the component and the chapter number (e.g. *b2 Sensory functions and pain*), followed by the second level (e.g. *b210 Seeing functions*), the third level (e.g. *b2100 Visual acuity functions*) and the fourth level (e.g. *b21000 Binocular acuity of distant vision*).

To encourage the use of the ICF in clinical practice and research, so called ICF Core Sets have been developed. ICF Core Sets are lists of ICF categories that were chosen in a multi-stage consensus process on which aspects of functioning are relevant for patients in specific settings or with specific health conditions, integrating evidence from empirical studies and input from experts. The ICF Research Branch of WHO Collaborating Center of the Family of International Classifications (DIMDI, Germany) at the University of Munich, Germany together with the Classifications, Assessments and Survey (CAS) Team and its partner organizations, developed a total of seven ICF Core Sets for patients with rehabilitation needs in the acute and early post-acute situation. The development process of those ICF Core Sets is described elsewhere [18]. The Acute ICF Core Sets focus on patients in acute hospitals with neurological, cardiopulmonary and musculoskeletal conditions [19-21], the Post-Acute ICF Core Sets refer to patients with neurological, cardiopulmonary and musculoskeletal conditions and aged patients [22-25]. Due to the patient orientated and multidisciplinary approach in rehabilitation, categories contained in the Acute and Post-Acute ICF Core Sets might represent potential goals of nursing interventions.

### Method

LEP was designed to cover nursing interventions in several settings, from operating room to midwifery. Therefore, the LEP nursing interventions had to be selected due to their relevance in rehabilitation nursing. Two nurses with long-time practical expertise in rehabilitation (MM, CB) independently judged whether the LEP nursing interventions were relevant for patients undergoing rehabilitation in the acute or early post-acute situation. After this the obtained results were compared. The final decision for including or excluding a LEP nursing intervention required a consensus between both experts. Results which had not been rated consensually had to be discussed until the experts agreed. To avoid misinterpretation of the concepts of LEP nursing interventions, the obtained selection had to be confirmed by a member of the LEP development team.

For the purpose of the study, the ICF Core Set categories of the seven acute and post-acute ICF Core Sets were merged to get a comprehensive selection of potential nursing intervention goals in the situation of acute and early post-acute rehabilitation. This reflects the fact that nurses in the acute and early post-acute situation care for patients with a variety of medical diagnoses [1]. This list of ICF categories consists of 62 ICF categories of the component "Body Functions", 17 ICF categories of the component "Body Structures" and 42 ICF categories of the component "Activities and Participation". Categories out of the component "Environmental Factors" were excluded from this study, because they rather interact with functioning, either as barriers or facilitators, than represent concepts of functioning as goals of nursing interventions [11].

A LEP nursing intervention does not explicitly describe the goal which could be achieved when performing an intervention. Nevertheless, one can assume that each nursing intervention aims to influence patients' functioning. Therefore, the ICF, which describes functioning, was used to describe potential goals of nursing interventions. The procedure to identify potential goals of LEP nursing interventions follows the so-called ICF linking procedure developed by Cieza and colleagues [26]. Accordingly, two nurses with long-time practical experience in the acute and early post-acute rehabilitation and good knowledge in the ICF classification and model carried out the procedure (MM, CB). In a first step, both experts independently judged whether the concepts contained in the selected ICF categories represent potential goals of LEP nursing interventions. After this the obtained results were compared. The final decision for linking a LEP nursing intervention to an ICF category required a consensus between both experts. Combinations which had not been rated consensually had to be discussed until the experts agreed.

## Results

Forty-eight LEP nursing interventions were consensually attributed to cover relevant nursing interventions for patients undergoing rehabilitation in the acute and early post-acute situation by both nurses and the member of the LEP development team.

One hundred and seven out of the 121 ICF categories (88%) were linked with at least one LEP nursing intervention. Considering the ICF components level, 45 of 62 (73%) "Body Functions" categories, 36 of 42 (86%) "Activity and Participation" categories and 14 of 17 (82%) "Body Structures" categories were linked with at least one LEP nursing intervention.

The ICF categories most frequently linked with LEP nursing interventions of all ICF components were *Respiration functions *(b440), *Experience of self and time functions *(b180), *Orientation functions *(b114) and *Focusing attention *(d160) (see Additional file 1).

The three ICF categories most frequently linked with LEP nursing interventions of the component "Body Functions" were *Respiration functions *(b440), *Experience of self and time functions *(b180) and *Orientation functions *(b114) (see Additional file 2).

The three ICF categories most frequently linked with LEP nursing interventions of the component "Body Structures" were *Spinal cord and related structures *(s120), *Structure of shoulder region *(s720) and *Structure of areas of skin *(s810) (see Additional file 3).

The three ICF categories most frequently linked with LEP nursing interventions of the component "Activities and Participation" were *Focusing attention *(d160), *Carrying out daily routine *(d230) and *Other purposeful sensing *(d120) (see Additional file 4).

Sixteen ICF categories of the ICF Core Sets (13%) could not be linked with LEP nursing interventions (see Table 3).

**Table 3:** ICF categories not identified as goals of LEP nursing interventions

b210	Seeing function
b215	Functions of structures adjoining the eye
b230	Hearing functions
b340	Alternative vocalization functions
b430	Haematological system functions
b435	Immunological system functions
b540	General metabolic functions
b545	Water, mineral and electrolyte balance functions
s130	Structures of meninges
s530	Structure of stomach
d135	Rehearsing
d315	Communication with receiving nonverbal messages
d335	Producing nonverbal messages
d860	Basic economic transactions
d870	Economic self-sufficiency
d930	Religion and spirituality

Thirty-two of 48 (67%) LEP nursing interventions were linked with at least one ICF category (see Additional file 1).

The LEP nursing interventions which were linked with the highest number of different ICF-categories as potential goals of nursing interventions of all were "therapeutic intervention", "patient-nurse communication/information giving" and "mobilising" (see Additional file 1).

The LEP nursing interventions which were linked with the highest number of different ICF-categories of the component "Body Functions" were "therapeutic intervention", "mobilising" and "positioning" (see Additional file 2).

The nursing interventions which were linked with the highest number of different ICF-categories of the component "Body Structures" were "patient-nurse communication/information giving", "positioning" and "obtaining and fitting support aids"(see Additional file 3).

The nursing interventions which were linked with the highest number of different ICF-categories of the component "Activities and Participation" were "therapeutic intervention", "patient-nurse communication/information giving" and "personal hygiene/dressing" (see Additional file 4). Seventeen LEP nursing interventions could not be linked with any ICF category (see Table 4).

**Table 4:** LEP nursing interventions not addressing ICF categories

1:1 care	Administration/Coordination	Specimen (other)	Conference/Consultation with physician
Blood Sample	Looking for object	Injection	Interdisciplinary Care Conference
Administering medication orally/rectally/vaginally or elsewhere	Looking for patient	Inserting venous catheter	Nursing Documentation
Test by nurses	Monitoring	Ultrasound	Restraint measures

## Discussion

All in all, nearly 90% of the ICF categories which were identified as relevant aspects of functioning in the acute and early post-acute situation could also be linked with goals of nursing interventions. Therefore, the ICF categories and especially the ICF Core Sets are indeed highly relevant for nursing care.

Nursing interventions could be linked to a multitude of different aspects of functioning. However, some of these aspects were linked more frequently than others, particularly those related to respiration (*Respiration functions*, *Additional respiratory functions *and *Respiratory muscle functions)*, consciousness and perception (categories from the ICF domain *Mental functions*), pain (*Pain*) and skin (*Protective functions of the skin*, *Repair functions of the skin *and *Structures of areas of skin*).

Functions related to respiration were linked with 15 out of the 48 nursing interventions, e.g. exercising effective coughing (included in "therapeutic intervention"), adequate positioning ("positioning"), early mobilisation ("mobilising"), chest tapping ("respiration support") or patient education, predominantly undertaken in post-acute settings ("patient-nurse communication/information giving"). These interventions prevent pulmonary complications or enhance patients' respiration performance. They are of prime importance since respiration functions are often impaired in the acute situation or at risk for being compromised by prolonged immobility. Therefore, interventions regarding respiration prevent severe sequels resulting from impaired breathing and related respiratory functions [27-30]

Pain is linked with numerous interventions, ranging from "therapeutic intervention" to "mobilising", "positioning", "personal hygiene/dressing", "perception training" "compressions", "massage" and "elimination". The goal of these interventions is not only to treat and reduce pain, but also to prevent it, since pain is always a major concern for nurses in the acute care and therefore seen as a highly prevalent challenge [31,32]. Furthermore, pain is also one of the most frequently addressed goals of physical therapists in the acute situation [33].

Pressure ulcers and their prevention are the main challenges for nurses in hospital care and therefore must be treated by multimodal approaches [34-36]. Consequently, one could expect that categories related to the skin were frequently linked with LEP nursing interventions in the acute situation.

Surprisingly, ICF categories related to consciousness and perception are also common goals of nursing interventions. This might be due to the increasing relevance of therapeutic concepts in rehabilitation nursing. Both specialized rehabilitation nurses in early post-acute facilities and nurses at intensive care units have adopted and are meanwhile familiar with treatment concepts used by other health professions, e.g. Bobath's approach to treat patients with hemiplegia [37-39], Affolter's concept for people with brain injuries [40] or the Kinaesthetics concept [41]. All of those concepts assume that adequate perception is a precondition of human movement and more complex activities and should therefore be addressed prominently. Additionally, an impairment of consciousness, orientation and perception is common after an acute episode of illness or injury [42] and will be addressed by most of the interventions, even by those ostensibly aimed at self care.

Although a total of 16 ICF Categories of the ICF Core Sets could not be linked to LEP nursing interventions (see Table 3) these categories are nevertheless indispensable for nursing practice. ICF categories such as *Seeing functions*, *Hearing functions *or *Immunological system functions *are important for nursing in the acute situation and have to be compensated or taken into account. They are, however, not directly addressed by nursing interventions with the intention to be regained or improved, nor can their loss be compensated by nursing interventions. For instance, seeing functions can be at risk due to impaired eyelid closure. Adequate nursing interventions in relation to the underlying condition are occlusive dressings or using artificial tears which directly aim to protect the conjunctives (covered by *Structure of the eyeball*) and only indirectly seeing functions [43].

The fact that *Religion and spirituality *was not linked to a nursing intervention needs a special annotation. Some might argue that religious and spiritual needs should be addressed by nursing interventions, because fulfilling these needs and beliefs is highly relevant for many patients, especially in critical situations after an acute episode. We agree with this, however, the ICF category *Religion and spirituality *does not describe spiritual and religious beliefs, which are Personal Factors that are not covered by the ICF yet, but a participation issue in terms of engaging in activities or ceremonies, what not seems to be a realistic goal in the early situation. Nevertheless, participation in religious activities may be very relevant for some acute patients and should therefore be enabled as soon as practicable.

"Therapeutic intervention", „patient-nurse communication/information giving" and "mobilising" were the most versatile nursing interventions linked with the highest number of different ICF categories (see additional file 1). By definition of the LEP, "Therapeutic intervention" includes nursing interventions additionally performed besides the routine interventions with therapeutic intentions [44]. These address the training of specific abilities, e.g. swallowing in patients with dysphagia, recapitulating lessons from speech or language therapy, or transferring from bed to the chair. „Patient-nurse communication/information giving" comprises each nursing intervention which seeks to inform or teach patients concerning their situation. Informing patients about their situation is essential at any time in hospital. Furthermore, teaching patients how to manage their new situation is of utmost importance for their recovery and rehabilitation, especially in the early post-acute situation and therefore might even influence a multitude of different aspects of functioning [45]. Our finding, that "mobilising" is able to address several aspects of functioning is also in line with the literature [10,27].

Even though 17 LEP nursing interventions (see Table 4) could not be directly linked with patients functioning in terms of ICF categories, these are important for the caring process and therefore for patient outcomes. Some of these nursing interventions concern the field of team-communication ("Administration/Coordination", "Conference/Consultation with physician", and "Interdisciplinary Care Conference"), others are related to getting blood samples and conducting other kinds of tests. In our opinion, interventions concerning the administration of medication ("Inserting venous catheter", "Administering medication orally/rectally/vaginally or elsewhere", and "Injection") do not influence patients' functioning. This might seem counterintuitive. However, the effects of drugs and medical products – albeit influencing functioning – are quite distinct from the effects of drug application and ought not to be attributed to application.

Our findings confirm the results of a previous study insofar as nursing interventions could be linked to the ICF [15]. However, in contrast to our study, the nursing interventions were derived from a different set of documentation terminology, and mainly focused on activities of daily living [46]. Thus, the results are not comparable.

Our study has some limitations. The two experts carried out an integrated two-step linking process. The first step was to derive goals of interventions from their practical knowledge, the second step was to link these goals to the most appropriate ICF category. The linking process is straightforward and an established procedure. Goal definition, however, is subjective and depends on professional training and experience. Nevertheless, although our approach might be only an approximation, results are supported by the literature, indicating that there is general consensus on the goals of nursing interventions.

Another limitation pertains to the LEP nursing interventions catalogue. It is a comprehensive workload classification, but not scientifically evaluated regarding its comprehensiveness. Yet, it has been reported to be practical and feasible [7], and it is used in a wide variety of settings by numerous institutions [8].

## Conclusion

The ICF Core Sets for the acute hospital and early post-acute rehabilitation facilities are highly relevant for rehabilitation nursing and can be an important tool to analyse nursing both in research and practice.

The systematic way of analysing nursing interventions with ICF Core Set categories indicates that nursing in the acute situation deals with far more complex tasks than the compensation of deficits in self care, the application of drugs and monitoring vital signs. Our results support the idea that nursing is concerned with functioning and should thus be seen as therapeutic.

As the ICF is designed to be understood by all involved groups, from patients to health professionals, the use of ICF Core Set categories to describe nursing intervention goals can be useful in two aspects. First, the ICF Core Sets enables nurses to describe their goals in a commonly understandable way. Thus the ICF has the potential to optimise the management of the rehabilitation process. Second, ICF Core Sets facilitate to consider patients' needs and wishes. The ICF may thus be a useful framework to set nursing intervention goals.

## Competing interests

The author(s) declare that they have no competing interests.

## Authors' contributions

MM, CB, and EG designed the study, MM and CB carried out the linking procedure, MM, CB, and RS analysed the data, MM prepared the manuscript. CB, RS, and EG assisted with data analysis and interpretation and revised the manuscript. GS supervised the study. All authors read and approved the final manuscript.

## Pre-publication history

The pre-publication history for this paper can be accessed here:



## Supplementary Material

Additional file 1 ICF categories (of all components) identified as goals of LEP nursing interventions. The table provided presents the results of the linking procedure for all ICF components. Click here for file

Additional file 2 ICF categories of the component Body Functions identified as goals of LEP nursing interventions. The table provided presents the results of the linking procedure for the ICF component Body Functions. Click here for file

Additional file 3 ICF categories of the component Body Structures identified as goals of LEP nursing interventions. The table provided presents the results of the linking procedure for the ICF component Body Structures. Click here for file

Additional file 4 ICF categories of the component Activities and Participation identified as goals of LEP nursing interventions. The table provided presents the results of the linking procedure for the ICF component Activities and Participation. Click here for file
