# Supplementary material for: Identification of ICF categories relevant for nursing in the situation of acute and early post-acute rehabilitation
Source: BMC Nurs. 2008 Feb 18;7:3. doi: 10.1186/1472-6955-7-3 (PMC2276191; doi:10.1186/1472-6955-7-3)
Supplement: Additional file 1 — ICF categories (of all components) identified as goals of LEP nursing interventions. The table provided presents the results of the linking procedure for all ICF components. [file 1472-6955-7-3-S1.doc]

**Additional File 1: ICF categories (of all components) identified as goals of LEP nursing interventions**

|  |  | LEP nursing interventions addressing ICF categories | | | | | | | | | | | | | | | | | | | | | | | | | | | | | | | |
| --- | --- | --- | --- | --- | --- | --- | --- | --- | --- | --- | --- | --- | --- | --- | --- | --- | --- | --- | --- | --- | --- | --- | --- | --- | --- | --- | --- | --- | --- | --- | --- | --- | --- |
| Linked ICF categories as goals of LEP nursing interventions | Number of linked interventions | Therapeutic Intervention | Patient-nurse communication/ information-giving | Mobilising | Positioning | Personal Hygiene / Dressing | Compressions | Massage | Perceptual training | Eating / Drinking | Obtaining and fitting support aids | Elimination | Activity and Recreation | Occupational Therapy | Wound Dressing / Wound Care | Nursing Visit | respiratory support | Bed Preparation | Physician Support | Drainage / Irrigation | Inserting catheter/tube | Oral / nasal / tracheal suctioning | Inhalation | cardiac support | Escort | Extubation | Case conference | Infusion | Intubation | Isolation procedures | Oxygen therapy | Technical Procedure | Tube change |
| b440 Respiration functions | 15 (31%) | x | x | x | x |  | x | x |  |  |  |  |  |  | x |  | x |  |  | x |  | x | x |  |  | x |  |  | x |  | x |  | x |
| b180 Experience of self and time functions | 13 (27%) | x | x | x | x | x |  | x | x | x |  | x | x | x |  | x | x |  |  |  |  |  |  |  |  |  |  |  |  |  |  |  |  |
| b114 Orientation functions | 12 (25%) | x | x | x |  | x | x | x | x | x |  | x | x | x |  | x |  |  |  |  |  |  |  |  |  |  |  |  |  |  |  |  |  |
| d160 Focusing attention | 12 (25%) | x | x | x | x | x | x | x | x | x |  | x | x |  |  | x |  |  |  |  |  |  |  |  |  |  |  |  |  |  |  |  |  |
| b140 Attention functions | 11 (23%) | x | x | x | x | x | x | x | x | x |  | x |  | x |  |  |  |  |  |  |  |  |  |  |  |  |  |  |  |  |  |  |  |
| b156 Perceptual functions | 11 (23%) | x | x | x | x | x | x | x | x | x |  | x | x |  |  |  |  |  |  |  |  |  |  |  |  |  |  |  |  |  |  |  |  |
| b280 Sensation of pain | 11 (23%) |  |  | x | x | x | x | x | x | x |  | x |  |  | x |  | x | x |  |  |  |  |  |  |  |  |  |  |  |  |  |  |  |
| b130 Energy and drive functions | 10 (21%) | x | x | x |  | x | x | x |  | x |  |  | x | x |  | x |  |  |  |  |  |  |  |  |  |  |  |  |  |  |  |  |  |
| b152 Emotional functions | 10 (21%) | x | x | x |  | x | x | x | x | x |  |  | x | x |  |  |  |  |  |  |  |  |  |  |  |  |  |  |  |  |  |  |  |
| b144 Memory functions | 9 (19%) | x | x | x |  | x |  |  | x | x |  | x | x | x |  |  |  |  |  |  |  |  |  |  |  |  |  |  |  |  |  |  |  |
| b147 Psychomotor functions | 9 (19%) | x |  | x | x | x | x | x | x | x |  |  | x |  |  |  |  |  |  |  |  |  |  |  |  |  |  |  |  |  |  |  |  |
| b450 Additional respiratory functions | 9 (19%) | x | x | x | x |  | x |  |  | x |  |  |  |  |  |  | x |  |  |  |  | x | x |  |  |  |  |  |  |  |  |  |  |
| b810 Protective functions of the skin | 9 (19%) |  | x | x | x | x |  |  |  |  | x | x |  |  | x |  |  | x | x |  |  |  |  |  |  |  |  |  |  |  |  |  |  |
| b820 Repair functions of the skin | 9 (19%) |  | x | x | x |  |  |  |  |  | x |  |  |  | x |  |  | x | x |  |  |  |  |  |  |  |  |  |  | x |  | x |  |
| d230 Carrying out daily routine | 9 (19%) |  | x | x |  | x |  |  |  | x |  | x | x | x |  | x |  |  |  |  |  |  |  |  | x |  |  |  |  |  |  |  |  |
| b110 Consciousness functions | 8 (17%) | x |  |  | x | x | x | x | x |  |  |  | x | x |  |  |  |  |  |  |  |  |  |  |  |  |  |  |  |  |  |  |  |
| b134 Sleep functions | 8 (17%) | x |  | x |  | x | x | x |  |  |  |  | x | x |  |  |  | x |  |  |  |  |  |  |  |  |  |  |  |  |  |  |  |
| b164 Higher-level cognitive functions | 8 (17%) | x | x | x |  | x |  |  | x | x |  | x |  | x |  |  |  |  |  |  |  |  |  |  |  |  |  |  |  |  |  |  |  |
| b260 Proprioceptive function | 8 (17%) | x |  | x | x | x |  | x | x | x | x |  |  |  |  |  |  |  |  |  |  |  |  |  |  |  |  |  |  |  |  |  |  |
| b460 Sensations associated with cardiovascular and respiratory functions | 8 (17%) |  | x | x | x |  | x | x |  |  |  |  |  |  |  |  | x |  |  |  |  | x | x |  |  |  |  |  |  |  |  |  |  |
| b735 Muscle tone functions | 8 (17%) | x | x | x | x | x | x | x |  |  | x |  |  |  |  |  |  |  |  |  |  |  |  |  |  |  |  |  |  |  |  |  |  |
| b176 Mental function of sequencing complex movements | 7 (15%) | x |  | x | x | x |  |  | x | x |  |  | x |  |  |  |  |  |  |  |  |  |  |  |  |  |  |  |  |  |  |  |  |
| b840 Sensation related to the skin | 7 (15%) |  |  | x | x | x | x |  |  |  |  |  |  |  | x |  |  | x | x |  |  |  |  |  |  |  |  |  |  |  |  |  |  |
| d120 Other purposeful sensing | 7 (15%) | x |  | x | x | x |  | x | x | x |  |  |  |  |  |  |  |  |  |  |  |  |  |  |  |  |  |  |  |  |  |  |  |
| b240 Sensations associated with hearing and vestibular function | 6 (13%) | x |  | x | x |  | x | x | x |  |  |  |  |  |  |  |  |  |  |  |  |  |  |  |  |  |  |  |  |  |  |  |  |
| b445 Respiratory muscle functions | 6 (13%) | x | x | x | x |  |  | x |  |  |  |  |  |  |  |  | x |  |  |  |  |  |  |  |  |  |  |  |  |  |  |  |  |
| b535 Sensations associated with the digestive system | 6 (13%) |  |  |  | x |  | x | x |  | x |  |  |  |  |  |  |  |  |  | x | x |  |  |  |  |  |  |  |  |  |  |  |  |
| b710 Mobility of joint functions | 6 (13%) | x | x | x | x | x | x |  |  |  |  |  |  |  |  |  |  |  |  |  |  |  |  |  |  |  |  |  |  |  |  |  |  |
| d570 Looking after one’s health | 6 (13%) |  | x | x | x | x |  |  |  | x |  |  |  |  |  | x |  |  |  |  |  |  |  |  |  |  |  |  |  |  |  |  |  |
| s120 Spinal cord and related structures | 6 (13%) |  | x |  | x |  |  |  |  |  | x |  |  |  |  |  |  | x | x |  |  |  |  |  | x |  |  |  |  |  |  |  |  |
| s720 Structure of shoulder region | 6 (13%) |  | x |  | x |  | x |  |  |  | x |  |  |  | x |  |  |  | x |  |  |  |  |  |  |  |  |  |  |  |  |  |  |
| b167 Mental functions of language | 5 (10%) | x | x |  |  |  |  |  | x |  |  |  |  | x |  | x |  |  |  |  |  |  |  |  |  |  |  |  |  |  |  |  |  |
| b415 Blood vessel functions | 5 (10%) |  |  | x | x | x | x |  |  |  |  |  |  |  | x |  |  |  |  |  |  |  |  |  |  |  |  |  |  |  |  |  |  |
| b420 Blood pressure functions | 5 (10%) |  |  | x | x | x | x |  |  |  |  |  |  |  |  |  |  |  |  |  |  |  |  | x |  |  |  |  |  |  |  |  |  |
| b455 Exercise tolerance functions | 5 (10%) | x |  | x | x | x |  |  |  |  |  |  |  |  |  |  | x |  |  |  |  |  |  |  |  |  |  |  |  |  |  |  |  |
| b525 Defecation functions | 5 (10%) |  | x |  |  |  | x |  |  |  |  | x |  |  |  |  |  |  |  | x | x |  |  |  |  |  |  |  |  |  |  |  |  |
| b760 Control of voluntary movement functions | 5 (10%) | x |  | x | x | x |  |  |  | x |  |  |  |  |  |  |  |  |  |  |  |  |  |  |  |  |  |  |  |  |  |  |  |
| b780 Sensations related to muscles and movement functions | 5 (10%) | x |  | x | x |  | x | x |  |  |  |  |  |  |  |  |  |  |  |  |  |  |  |  |  |  |  |  |  |  |  |  |  |
| d177 Making decisions | 5 (10%) |  | x |  |  | x |  |  |  | x |  | x |  |  |  | x |  |  |  |  |  |  |  |  |  |  |  |  |  |  |  |  |  |
| d240 Handling stress and other psychological demands | 5 (10%) |  | x |  |  |  | x | x |  |  |  |  |  |  |  | x |  |  | x |  |  |  |  |  |  |  |  |  |  |  |  |  |  |
| d445 Hand and arm use | 5 (10%) | x |  | x | x | x |  |  |  | x |  |  |  |  |  |  |  |  |  |  |  |  |  |  |  |  |  |  |  |  |  |  |  |
| s810 Structure of areas of skin | 5 (10%) |  |  |  | x | x |  |  |  |  |  |  |  |  | x |  |  | x | x |  |  |  |  |  |  |  |  |  |  |  |  |  |  |
| b265 Touch function | 4 (8%) | x |  |  |  | x |  | x | x |  |  |  |  |  |  |  |  |  |  |  |  |  |  |  |  |  |  |  |  |  |  |  |  |
| b510 Ingestion functions | 4 (8%) | x | x |  |  |  |  |  |  | x |  |  |  |  |  |  |  |  |  |  | x |  |  |  |  |  |  |  |  |  |  |  |  |
| b620 Urination functions | 4 (8%) |  | x |  |  |  | x |  |  |  |  | x |  |  |  |  |  |  |  |  | x |  |  |  |  |  |  |  |  |  |  |  |  |
| b630 Sensations associated with urinary functions | 4 (8%) |  |  |  |  |  | x |  |  |  |  | x |  |  |  |  |  |  |  | x | x |  |  |  |  |  |  |  |  |  |  |  |  |
| b715 Stability of joint functions | 4 (8%) |  |  | x | x |  |  |  |  |  | x |  |  |  | x |  |  |  |  |  |  |  |  |  |  |  |  |  |  |  |  |  |  |
| b730 Muscle power functions | 4 (8%) | x |  | x |  | x |  |  |  |  | x |  |  |  |  |  |  |  |  |  |  |  |  |  |  |  |  |  |  |  |  |  |  |
| b740 Muscle endurance functions | 4 (8%) | x |  | x |  | x |  |  |  |  | x |  |  |  |  |  |  |  |  |  |  |  |  |  |  |  |  |  |  |  |  |  |  |
| b770 Gait pattern functions | 4 (8%) | x | x | x |  |  |  |  |  |  | x |  |  |  |  |  |  |  |  |  |  |  |  |  |  |  |  |  |  |  |  |  |  |
| d410 Changing basic body position | 4 (8%) | x |  | x | x | x |  |  |  |  |  |  |  |  |  |  |  |  |  |  |  |  |  |  |  |  |  |  |  |  |  |  |  |
| d415 Maintaining a body position | 4 (8%) | x |  | x | x | x |  |  |  |  |  |  |  |  |  |  |  |  |  |  |  |  |  |  |  |  |  |  |  |  |  |  |  |
| d420 Transferring oneself | 4 (8%) | x |  | x | x | x |  |  |  |  |  |  |  |  |  |  |  |  |  |  |  |  |  |  |  |  |  |  |  |  |  |  |  |
| d450 Walking | 4 (8%) | x |  | x |  | x |  |  |  |  | x |  |  |  |  |  |  |  |  |  |  |  |  |  |  |  |  |  |  |  |  |  |  |
| d550 Eating | 4 (8%) | x | x |  |  |  |  |  |  | x | x |  |  |  |  |  |  |  |  |  |  |  |  |  |  |  |  |  |  |  |  |  |  |
| d560 Drinking | 4 (8%) | x | x |  |  |  |  |  |  | x | x |  |  |  |  |  |  |  |  |  |  |  |  |  |  |  |  |  |  |  |  |  |  |
| s710 Structure of head and neck region | 4 (8%) |  | x | x | x |  |  |  |  |  | x |  |  |  |  |  |  |  |  |  |  |  |  |  |  |  |  |  |  |  |  |  |  |
| s730 Structure of upper extremity | 4 (8%) |  | x |  | x |  | x |  |  |  | x |  |  |  |  |  |  |  |  |  |  |  |  |  |  |  |  |  |  |  |  |  |  |
| s760 Structure of trunk | 4 (8%) |  | x | x | x |  |  |  |  |  | x |  |  |  |  |  |  |  |  |  |  |  |  |  |  |  |  |  |  |  |  |  |  |
| b117 Intellectual functions | 3 (6%) | x | x |  |  |  |  |  | x |  |  |  |  |  |  |  |  |  |  |  |  |  |  |  |  |  |  |  |  |  |  |  |  |
| b235 Vestibular functions | 3 (6%) | x |  |  |  |  |  | x | x |  |  |  |  |  |  |  |  |  |  |  |  |  |  |  |  |  |  |  |  |  |  |  |  |
| b270 Sensory functions related to temperature and other stimuli | 3 (6%) | x |  |  |  |  |  | x | x |  |  |  |  |  |  |  |  |  |  |  |  |  |  |  |  |  |  |  |  |  |  |  |  |
| b410 Heart functions | 3 (6%) |  |  | x | x |  |  |  |  |  |  |  |  |  |  |  |  |  |  |  |  |  |  | x |  |  |  |  |  |  |  |  |  |
| b515 Digestive functions | 3 (6%) |  |  |  | x |  | x | x |  |  |  |  |  |  |  |  |  |  |  |  |  |  |  |  |  |  |  |  |  |  |  |  |  |
| b530 Weight maintenance functions | 3 (6%) |  | x |  |  |  |  |  |  | x |  |  |  |  |  |  |  |  |  |  |  |  |  |  |  |  |  | x |  |  |  |  |  |
| b755 Involuntary movement reaction functions | 3 (6%) | x |  | x | x |  |  |  |  |  |  |  |  |  |  |  |  |  |  |  |  |  |  |  |  |  |  |  |  |  |  |  |  |
| d175 Solving problems | 3 (6%) | x | x |  |  |  |  |  | x |  |  |  |  |  |  |  |  |  |  |  |  |  |  |  |  |  |  |  |  |  |  |  |  |
| d440 Fine hand use | 3 (6%) | x |  |  |  | x |  |  |  | x |  |  |  |  |  |  |  |  |  |  |  |  |  |  |  |  |  |  |  |  |  |  |  |
| d460 Moving around in different locations | 3 (6%) | x |  | x |  |  |  |  |  |  | x |  |  |  |  |  |  |  |  |  |  |  |  |  |  |  |  |  |  |  |  |  |  |
| d465 Moving around using equipment | 3 (6%) | x |  | x |  |  |  |  |  |  | x |  |  |  |  |  |  |  |  |  |  |  |  |  |  |  |  |  |  |  |  |  |  |
| d510 Washing oneself | 3 (6%) | x | x |  |  | x |  |  |  |  |  |  |  |  |  |  |  |  |  |  |  |  |  |  |  |  |  |  |  |  |  |  |  |
| d520 Caring for body parts | 3 (6%) | x | x |  |  | x |  |  |  |  |  |  |  |  |  |  |  |  |  |  |  |  |  |  |  |  |  |  |  |  |  |  |  |
| d530 Toileting | 3 (6%) |  | x |  |  |  |  |  |  |  | x | x |  |  |  |  |  |  |  |  |  |  |  |  |  |  |  |  |  |  |  |  |  |
| d540 Dressing | 3 (6%) | x | x |  |  | x |  |  |  |  |  |  |  |  |  |  |  |  |  |  |  |  |  |  |  |  |  |  |  |  |  |  |  |
| s320 Structure of mouth | 3 (6%) |  | x |  |  | x |  |  |  |  |  |  |  |  | x |  |  |  |  |  |  |  |  |  |  |  |  |  |  |  |  |  |  |
| s750 Structure of lower extremity | 3 (6%) |  | x |  | x |  |  |  |  |  | x |  |  |  |  |  |  |  |  |  |  |  |  |  |  |  |  |  |  |  |  |  |  |
| b550 Thermoregulatory functions | 2 (4%) |  |  |  |  | x | x |  |  |  |  |  |  |  |  |  |  |  |  |  |  |  |  |  |  |  |  |  |  |  |  |  |  |
| b765 Involuntary movement functions | 2 (4%) | x |  |  |  |  |  | x |  |  |  |  |  |  |  |  |  |  |  |  |  |  |  |  |  |  |  |  |  |  |  |  |  |
| d110 Watching | 2 (4%) |  |  |  |  |  |  |  | x |  |  |  | x |  |  |  |  |  |  |  |  |  |  |  |  |  |  |  |  |  |  |  |  |
| d166 Reading | 2 (4%) | x |  |  |  |  |  |  | x |  |  |  |  |  |  |  |  |  |  |  |  |  |  |  |  |  |  |  |  |  |  |  |  |
| d170 Writing | 2 (4%) | x |  |  |  |  |  |  | x |  |  |  |  |  |  |  |  |  |  |  |  |  |  |  |  |  |  |  |  |  |  |  |  |
| d310 Communicating with - receiving - spoken messages | 2 (4%) |  | x |  |  |  |  |  | x |  |  |  |  |  |  |  |  |  |  |  |  |  |  |  |  |  |  |  |  |  |  |  |  |
| d360 Using communication devices and techniques | 2 (4%) | x | x |  |  |  |  |  |  |  |  |  |  |  |  |  |  |  |  |  |  |  |  |  |  |  |  |  |  |  |  |  |  |
| d430 Lifting and carrying objects | 2 (4%) | x |  | x |  |  |  |  |  |  |  |  |  |  |  |  |  |  |  |  |  |  |  |  |  |  |  |  |  |  |  |  |  |
| d910 Community life | 2 (4%) |  |  | x |  | x |  |  |  |  |  |  |  |  |  |  |  |  |  |  |  |  |  |  |  |  |  |  |  |  |  |  |  |
| s110 Structure of brain | 2 (4%) |  |  |  | x |  |  |  |  |  |  |  |  |  |  |  |  |  |  | x |  |  |  |  |  |  |  |  |  |  |  |  |  |
| s610 Structure of urinary system | 2 (4%) |  |  |  |  |  |  |  |  |  |  |  |  |  |  |  |  |  |  | x | x |  |  |  |  |  |  |  |  |  |  |  |  |
| s740 Structure of pelvic region | 2 (4%) |  | x |  | x |  |  |  |  |  |  |  |  |  |  |  |  |  |  |  |  |  |  |  |  |  |  |  |  |  |  |  |  |
| b126 Temperament and personality functions | 1 (2%) |  |  |  |  |  |  |  |  |  |  |  |  | x |  |  |  |  |  |  |  |  |  |  |  |  |  |  |  |  |  |  |  |
| b160 Thought functions | 1 (2%) |  |  |  |  |  |  |  | x |  |  |  |  |  |  |  |  |  |  |  |  |  |  |  |  |  |  |  |  |  |  |  |  |
| b310 Voice functions | 1 (2%) | x |  |  |  |  |  |  |  |  |  |  |  |  |  |  |  |  |  |  |  |  |  |  |  |  |  |  |  |  |  |  |  |
| b320 Articulation functions | 1 (2%) | x |  |  |  |  |  |  |  |  |  |  |  |  |  |  |  |  |  |  |  |  |  |  |  |  |  |  |  |  |  |  |  |
| b610 Urinary excretory functions | 1 (2%) |  |  |  |  |  |  | x |  |  |  |  |  |  |  |  |  |  |  |  |  |  |  |  |  |  |  |  |  |  |  |  |  |
| d115 Listening | 1 (2%) |  |  |  |  |  |  |  | x |  |  |  |  |  |  |  |  |  |  |  |  |  |  |  |  |  |  |  |  |  |  |  |  |
| d130 Copying | 1 (2%) | x |  |  |  |  |  |  |  |  |  |  |  |  |  |  |  |  |  |  |  |  |  |  |  |  |  |  |  |  |  |  |  |
| d155 Acquiring skills | 1 (2%) | x |  |  |  |  |  |  |  |  |  |  |  |  |  |  |  |  |  |  |  |  |  |  |  |  |  |  |  |  |  |  |  |
| d330 Speaking | 1 (2%) | x |  |  |  |  |  |  |  |  |  |  |  |  |  |  |  |  |  |  |  |  |  |  |  |  |  |  |  |  |  |  |  |
| d350 Conversation | 1 (2%) |  | x |  |  |  |  |  |  |  |  |  |  |  |  |  |  |  |  |  |  |  |  |  |  |  |  |  |  |  |  |  |  |
| d760 Family relationships | 1 (2%) |  | x |  |  |  |  |  |  |  |  |  |  |  |  |  |  |  |  |  |  |  |  |  |  |  |  |  |  |  |  |  |  |
| d770 Intimate relationships | 1 (2%) |  | x |  |  |  |  |  |  |  |  |  |  |  |  |  |  |  |  |  |  |  |  |  |  |  |  |  |  |  |  |  |  |
| d940 Human rights | 1 (2%) |  |  |  |  |  |  |  |  |  |  |  |  |  |  |  |  |  |  |  |  |  |  |  |  |  | x |  |  |  |  |  |  |
| s410 Structure of cardiovascular system | 1 (2%) |  | x |  |  |  |  |  |  |  |  |  |  |  |  |  |  |  |  |  |  |  |  |  |  |  |  |  |  |  |  |  |  |
| s430 Structure of respiratory system | 1 (2%) |  |  |  |  |  |  |  |  |  |  |  |  |  |  |  | x |  |  |  |  |  |  |  |  |  |  |  |  |  |  |  |  |
| s620 Structure of pelvic floor | 1 (2%) |  | x |  |  |  |  |  |  |  |  |  |  |  |  |  |  |  |  |  |  |  |  |  |  |  |  |  |  |  |  |  |  |
| s770 Additional musculoskeletal structures related to movement | 1 (2%) |  | x |  |  |  |  |  |  |  |  |  |  |  |  |  |  |  |  |  |  |  |  |  |  |  |  |  |  |  |  |  |  |
