# Supplementary material for: Identification of ICF categories relevant for nursing in the situation of acute and early post-acute rehabilitation
Source: BMC Nurs. 2008 Feb 18;7:3. doi: 10.1186/1472-6955-7-3 (PMC2276191; doi:10.1186/1472-6955-7-3)
Supplement: Additional file 2 — ICF categories of the component Body Functions identified as goals of LEP nursing interventions. The table provided presents the results of the linking procedure for the ICF component Body Functions. [file 1472-6955-7-3-S2.doc]

**Additional File 2: ICF categories of the component Body Functions identified as goals of LEP nursing interventions**

|  |  | LEP nursing interventions addressing ICF categories | | | | | | | | | | | | | | | | | | | | | | | | | | | | | |
| --- | --- | --- | --- | --- | --- | --- | --- | --- | --- | --- | --- | --- | --- | --- | --- | --- | --- | --- | --- | --- | --- | --- | --- | --- | --- | --- | --- | --- | --- | --- | --- |
| Linked ICF categories as goals of LEP nursing  interventions | number of linked interventions | Therapeutic Intervention | Mobilising | Positioning | Personal Hygiene/Dressing | Compressions | Massage | Patient-nurse communication/  information-giving | Perceptual training | Eating / Drinking | Elimination | Occupational Therapy | Activity and Recreation | Obtaining and fitting support aids | Wound Dressing / Wound Care | respiratory support | Bed Preparation | Inserting catheter/tube | Nursing Visit | Drainage / Irrigation | Physician Support | Oral / nasal / tracheal suctioning | Inhalation | cardiac support | Extubation | Infusion | Intubation | Isolation procedures | Oxygen therapy | Technical Procedure | Tube change |
| b440 Respiration functions | 15 (31%) | x | x | x |  | x | x | x |  |  |  |  |  |  | x | x |  |  |  | x |  | x | x |  | x |  | x |  | x |  | x |
| b180 Experience of self and time functions | 13 (27%) | x | x | x | x |  | x | x | x | x | x | x | x |  |  | x |  |  | x |  |  |  |  |  |  |  |  |  |  |  |  |
| b114 Orientation functions | 12 (25%) | x | x |  | x | x | x | x | x | x | x | x | x |  |  |  |  |  | x |  |  |  |  |  |  |  |  |  |  |  |  |
| b140 Attention functions | 11 (23%) | x | x | x | x | x | x | x | x | x | x | x |  |  |  |  |  |  |  |  |  |  |  |  |  |  |  |  |  |  |  |
| b156 Perceptual functions | 11 (23%) | x | x | x | x | x | x | x | x | x | x |  | x |  |  |  |  |  |  |  |  |  |  |  |  |  |  |  |  |  |  |
| b280 Sensation of pain | 11 (23%) |  | x | x | x | x | x |  | x | x | x |  |  |  | x | x | x |  |  |  |  |  |  |  |  |  |  |  |  |  |  |
| b130 Energy and drive functions | 10 (21%) | x | x |  | x | x | x | x |  | x |  | x | x |  |  |  |  |  | x |  |  |  |  |  |  |  |  |  |  |  |  |
| b152 Emotional functions | 10 (21%) | x | x |  | x | x | x | x | x | x |  | x | x |  |  |  |  |  |  |  |  |  |  |  |  |  |  |  |  |  |  |
| b144 Memory functions | 9 (19%) | x | x |  | x |  |  | x | x | x | x | x | x |  |  |  |  |  |  |  |  |  |  |  |  |  |  |  |  |  |  |
| b147 Psychomotor functions | 9 (19%) | x | x | x | x | x | x |  | x | x |  |  | x |  |  |  |  |  |  |  |  |  |  |  |  |  |  |  |  |  |  |
| b450 Additional respiratory functions | 9 (19%) | x | x | x |  | x |  | x |  | x |  |  |  |  |  | x |  |  |  |  |  | x | x |  |  |  |  |  |  |  |  |
| b810 Protective functions of the skin | 9 (19%) |  | x | x | x |  |  | x |  |  | x |  |  | x | x |  | x |  |  |  | x |  |  |  |  |  |  |  |  |  |  |
| b820 Repair functions of the skin | 9 (19%) |  | x | x |  |  |  | x |  |  |  |  |  | x | x |  | x |  |  |  | x |  |  |  |  |  |  | x |  | x |  |
| b110 Consciousness functions | 8 (17%) | x |  | x | x | x | x |  | x |  |  | x | x |  |  |  |  |  |  |  |  |  |  |  |  |  |  |  |  |  |  |
| b134 Sleep functions | 8 (17%) | x | x |  | x | x | x |  |  |  |  | x | x |  |  |  | x |  |  |  |  |  |  |  |  |  |  |  |  |  |  |
| b164 Higher-level cognitive functions | 8 (17%) | x | x |  | x |  |  | x | x | x | x | x |  |  |  |  |  |  |  |  |  |  |  |  |  |  |  |  |  |  |  |
| b260 Proprioceptive functions | 8 (17%) | x | x | x | x |  | x |  | x | x |  |  |  | x |  |  |  |  |  |  |  |  |  |  |  |  |  |  |  |  |  |
| b460 Sensations associated with cardiovasculary and respiratory functions | 8 (17%) |  | x | x |  | x | x | x |  |  |  |  |  |  |  | x |  |  |  |  |  | x | x |  |  |  |  |  |  |  |  |
| b735 Muscle tone functions | 8 (17%) | x | x | x | x | x | x | x |  |  |  |  |  | x |  |  |  |  |  |  |  |  |  |  |  |  |  |  |  |  |  |
| b176 Mental function of sequencing complex movements | 7 (15%) | x | x | x | x |  |  |  | x | x |  |  | x |  |  |  |  |  |  |  |  |  |  |  |  |  |  |  |  |  |  |
| b840 Sensation related to the skin | 7 (15%) |  | x | x | x | x |  |  |  |  |  |  |  |  | x |  | x |  |  |  | x |  |  |  |  |  |  |  |  |  |  |
| b240 Sensations ass. with hearing and vestibular function | 6 (13%) | x | x | x |  | x | x |  | x |  |  |  |  |  |  |  |  |  |  |  |  |  |  |  |  |  |  |  |  |  |  |
| b445 Respiratory muscle functions | 6 (13%) | x | x | x |  |  | x | x |  |  |  |  |  |  |  | x |  |  |  |  |  |  |  |  |  |  |  |  |  |  |  |
| b535 Sensations associated with the digestive system | 6 (13%) |  |  | x |  | x | x |  |  | x |  |  |  |  |  |  |  | x |  | x |  |  |  |  |  |  |  |  |  |  |  |
| b710 Mobility of joint functions | 6 (13%) | x | x | x | x | x |  | x |  |  |  |  |  |  |  |  |  |  |  |  |  |  |  |  |  |  |  |  |  |  |  |
| b167 Mental functions of language | 5 (10%) | x |  |  |  |  |  | x | x |  |  | x |  |  |  |  |  |  | x |  |  |  |  |  |  |  |  |  |  |  |  |
| b415 Blood vessel functions | 5 (10%) |  | x | x | x | x |  |  |  |  |  |  |  |  | x |  |  |  |  |  |  |  |  |  |  |  |  |  |  |  |  |
| b420 Blood pressure functions | 5 (10%) |  | x | x | x | x |  |  |  |  |  |  |  |  |  |  |  |  |  |  |  |  |  | x |  |  |  |  |  |  |  |
| b455 Exercise tolerance functions | 5 (10%) | x | x | x | x |  |  |  |  |  |  |  |  |  |  | x |  |  |  |  |  |  |  |  |  |  |  |  |  |  |  |
| b525 Defecation functions | 5 (10%) |  |  |  |  | x |  | x |  |  | x |  |  |  |  |  |  | x |  | x |  |  |  |  |  |  |  |  |  |  |  |
| b760 Control of voluntary movement functions | 5 (10%) | x | x | x | x |  |  |  |  | x |  |  |  |  |  |  |  |  |  |  |  |  |  |  |  |  |  |  |  |  |  |
| b780 Sensations rel. to muscles and movement functions | 5 (10%) | x | x | x |  | x | x |  |  |  |  |  |  |  |  |  |  |  |  |  |  |  |  |  |  |  |  |  |  |  |  |
| b265 Touch function | 4 (8%) | x |  |  | x |  | x |  | x |  |  |  |  |  |  |  |  |  |  |  |  |  |  |  |  |  |  |  |  |  |  |
| b510 Ingestion functions | 4 (8%) | x |  |  |  |  |  | x |  | x |  |  |  |  |  |  |  | x |  |  |  |  |  |  |  |  |  |  |  |  |  |
| b620 Urination functions | 4 (8%) |  |  |  |  | x |  | x |  |  | x |  |  |  |  |  |  | x |  |  |  |  |  |  |  |  |  |  |  |  |  |
| b630 Sensations associated with urinary functions | 4 (8%) |  |  |  |  | x |  |  |  |  | x |  |  |  |  |  |  | x |  | x |  |  |  |  |  |  |  |  |  |  |  |
| b715 Stability of joint functions | 4 (8%) |  | x | x |  |  |  |  |  |  |  |  |  | x | x |  |  |  |  |  |  |  |  |  |  |  |  |  |  |  |  |
| b730 Muscle power functions | 4 (8%) | x | x |  | x |  |  |  |  |  |  |  |  | x |  |  |  |  |  |  |  |  |  |  |  |  |  |  |  |  |  |
| b740 Muscle endurance functions | 4 (8%) | x | x |  | x |  |  |  |  |  |  |  |  | x |  |  |  |  |  |  |  |  |  |  |  |  |  |  |  |  |  |
| b770 Gait pattern functions | 4 (8%) | x | x |  |  |  |  | x |  |  |  |  |  | x |  |  |  |  |  |  |  |  |  |  |  |  |  |  |  |  |  |
| b117 Intellectual functions | 3 (6%) | x |  |  |  |  |  | x | x |  |  |  |  |  |  |  |  |  |  |  |  |  |  |  |  |  |  |  |  |  |  |
| b235 Vestibular functions | 3 (6%) | x |  |  |  |  | x |  | x |  |  |  |  |  |  |  |  |  |  |  |  |  |  |  |  |  |  |  |  |  |  |
| b270 Sensory functions related to temperature a. o. stimuli | 3 (6%) | x |  |  |  |  | x |  | x |  |  |  |  |  |  |  |  |  |  |  |  |  |  |  |  |  |  |  |  |  |  |
| b410 Heart functions | 3 (6%) |  | x | x |  |  |  |  |  |  |  |  |  |  |  |  |  |  |  |  |  |  |  | x |  |  |  |  |  |  |  |
| b515 Digestive functions | 3 (6%) |  |  | x |  | x | x |  |  |  |  |  |  |  |  |  |  |  |  |  |  |  |  |  |  |  |  |  |  |  |  |
| b530 Weight maintenance functions | 3 (6%) |  |  |  |  |  |  | x |  | x |  |  |  |  |  |  |  |  |  |  |  |  |  |  |  | x |  |  |  |  |  |
| b755 Involuntary movement reaction functions | 3 (6%) | x | x | x |  |  |  |  |  |  |  |  |  |  |  |  |  |  |  |  |  |  |  |  |  |  |  |  |  |  |  |
| b550 Thermoregulatory functions | 2 (4%) |  |  |  | x | x |  |  |  |  |  |  |  |  |  |  |  |  |  |  |  |  |  |  |  |  |  |  |  |  |  |
| b765 Involuntary movement functions | 2 (4%) | x |  |  |  |  | x |  |  |  |  |  |  |  |  |  |  |  |  |  |  |  |  |  |  |  |  |  |  |  |  |
| b126 Temperament and personality functions | 1 (2%) |  |  |  |  |  |  |  |  |  |  | x |  |  |  |  |  |  |  |  |  |  |  |  |  |  |  |  |  |  |  |
| b160 Thought functions | 1 (2%) |  |  |  |  |  |  |  | x |  |  |  |  |  |  |  |  |  |  |  |  |  |  |  |  |  |  |  |  |  |  |
| b310 Voice functions | 1 (2%) | x |  |  |  |  |  |  |  |  |  |  |  |  |  |  |  |  |  |  |  |  |  |  |  |  |  |  |  |  |  |
| b320 Articulation functions | 1 (2%) | x |  |  |  |  |  |  |  |  |  |  |  |  |  |  |  |  |  |  |  |  |  |  |  |  |  |  |  |  |  |
| b610 Urinary excretory functions | 1 (2%) |  |  |  |  |  | x |  |  |  |  |  |  |  |  |  |  |  |  |  |  |  |  |  |  |  |  |  |  |  |  |
