# Supplementary material for: Identification of ICF categories relevant for nursing in the situation of acute and early post-acute rehabilitation
Source: BMC Nurs. 2008 Feb 18;7:3. doi: 10.1186/1472-6955-7-3 (PMC2276191; doi:10.1186/1472-6955-7-3)
Supplement: Additional file 3 — ICF categories of the component Body Structures identified as goals of LEP nursing interventions. The table provided presents the results of the linking procedure for the ICF component Body Structures. [file 1472-6955-7-3-S3.doc]

**Additional File 3: ICF categories of the component Body Structures identified as goals of LEP nursing interventions**

|  |  | LEP nursing interventions addressing ICF categories | | | | | | | | | | | | |
| --- | --- | --- | --- | --- | --- | --- | --- | --- | --- | --- | --- | --- | --- | --- |
| Linked ICF categories as goals of LEP nursing  interventions | number of linked interventions | Patient-nurse communication/ information-giving | Positioning | Obtaining and fitting support aids | Wound Dressing / Wound Care | Physician Support | Mobilising | Personal Hygiene / Dressing | Compressions | Bed Preparation | Drainage / Irrigation | respiratory support | Inserting catheter/tube | Escort |
| s120 Spinal cord and related structures | 6 (13%) | x | x | x |  | x |  |  |  | x |  |  |  | x |
| s720 Structure of shoulder region | 6 (13%) | x | x | x | x | x |  |  | x |  |  |  |  |  |
| s810 Structure of areas of skin | 5 (10%) |  | x |  | x | x |  | x |  | x |  |  |  |  |
| s710 Structure of head and neck region | 4 (8%) | x | x | x |  |  | x |  |  |  |  |  |  |  |
| s730 Structure of upper extremity | 4 (8%) | x | x | x |  |  |  |  | x |  |  |  |  |  |
| s760 Structure of trunk | 4 (8%) | x | x | x |  |  | x |  |  |  |  |  |  |  |
| s320 Structure of mouth | 3 (6%) | x |  |  | x |  |  | x |  |  |  |  |  |  |
| s750 Structure of lower extremity | 3 (6%) | x | x | x |  |  |  |  |  |  |  |  |  |  |
| s110 Structure of brain | 2 (4%) |  | x |  |  |  |  |  |  |  | x |  |  |  |
| s610 Structure of urinary system | 2 (4%) |  |  |  |  |  |  |  |  |  | x |  | x |  |
| s740 Structure of pelvic region | 2 (4%) | x | x |  |  |  |  |  |  |  |  |  |  |  |
| s410 Structure of cardiovascular system | 1 (2%) | x |  |  |  |  |  |  |  |  |  |  |  |  |
| s430 Structure of respiratory system | 1 (2%) |  |  |  |  |  |  |  |  |  |  | x |  |  |
| s620 Structure of pelvic floor | 1 (2%) | x |  |  |  |  |  |  |  |  |  |  |  |  |
| s770 Additional musculoskeletal structures  related to movement | 1 (2%) | x |  |  |  |  |  |  |  |  |  |  |  |  |
