# Supplementary material for: Identification of ICF categories relevant for nursing in the situation of acute and early post-acute rehabilitation
Source: BMC Nurs. 2008 Feb 18;7:3. doi: 10.1186/1472-6955-7-3 (PMC2276191; doi:10.1186/1472-6955-7-3)
Supplement: Additional file 4 — ICF categories of the component Activities and Participation identified as goals of LEP nursing interventions. The table provided presents the results of the linking procedure for the ICF component Activities and Participation. [file 1472-6955-7-3-S4.doc]

**Additional File 4: ICF categories of the component Activities and Participation identified as goals of LEP nursing interventions**

|  |  | LEP nursing interventions addressing ICF categories | | | | | | | | | | | | | | | | |
| --- | --- | --- | --- | --- | --- | --- | --- | --- | --- | --- | --- | --- | --- | --- | --- | --- | --- | --- |
| Linked ICF categories as goals of LEP nursing interventions | number of linked interventions | Therapeutic Intervention | Patient-nurse communication | Personal Hygiene / Dressing | Mobilising | Eating / Drinking | Perceptual training | Positioning | Obtaining and fitting support aids | Nursing Visit | Elimination | Massage | Activity and Recreation | Compressions | Occupational Therapy | Physician Support | Escort | Case conference |
| d160 Focusing attention | 12 (25%) | x | x | x | x | x | x | x |  | x | x | x | x | x |  |  |  |  |
| d230 Carrying out daily routine | 9 (19%) |  | x | x | x | x |  |  |  | x | x |  | x |  | x |  | x |  |
| d120 Other purposeful sensing | 7 (15%) | x |  | x | x | x | x | x |  |  |  | x |  |  |  |  |  |  |
| d570 Looking after one’s health | 6 (13%) |  | x | x | x | x |  | x |  | x |  |  |  |  |  |  |  |  |
| d177 Making decisions | 5 (10%) |  | x | x |  | x |  |  |  | x | x |  |  |  |  |  |  |  |
| d240 Handling stress and other psychological demands | 5 (10%) |  | x |  |  |  |  |  |  | x |  | x |  | x |  | x |  |  |
| d445 Hand and arm use | 5 (10%) | x |  | x | x | x |  | x |  |  |  |  |  |  |  |  |  |  |
| d410 Changing basic body position | 4 (8%) | x |  | x | x |  |  | x |  |  |  |  |  |  |  |  |  |  |
| d415 Maintaining a body position | 4 (8%) | x |  | x | x |  |  | x |  |  |  |  |  |  |  |  |  |  |
| d420 Transferring oneself | 4 (8%) | x |  | x | x |  |  | x |  |  |  |  |  |  |  |  |  |  |
| d450 Walking | 4 (8%) | x |  | x | x |  |  |  | x |  |  |  |  |  |  |  |  |  |
| d550 Eating | 4 (8%) | x | x |  |  | x |  |  | x |  |  |  |  |  |  |  |  |  |
| d560 Drinking | 4 (8%) | x | x |  |  | x |  |  | x |  |  |  |  |  |  |  |  |  |
| d175 Solving problems | 3 (6%) | x | x |  |  |  | x |  |  |  |  |  |  |  |  |  |  |  |
| d440 Fine hand use | 3 (6%) | x |  | x |  | x |  |  |  |  |  |  |  |  |  |  |  |  |
| d460 Moving around in different locations | 3 (6%) | x |  |  | x |  |  |  | x |  |  |  |  |  |  |  |  |  |
| d465 Moving around using equipment | 3 (6%) | x |  |  | x |  |  |  | x |  |  |  |  |  |  |  |  |  |
| d510 Washing oneself | 3 (6%) | x | x | x |  |  |  |  |  |  |  |  |  |  |  |  |  |  |
| d520 Caring for body parts | 3 (6%) | x | x | x |  |  |  |  |  |  |  |  |  |  |  |  |  |  |
| d530 Toileting | 3 (6%) |  | x |  |  |  |  |  | x |  | x |  |  |  |  |  |  |  |
| d540 Dressing | 3 (6%) | x | x | x |  |  |  |  |  |  |  |  |  |  |  |  |  |  |
| d110 Watching | 2 (4%) |  |  |  |  |  | x |  |  |  |  |  | x |  |  |  |  |  |
| d166 Reading | 2 (4%) | x |  |  |  |  | x |  |  |  |  |  |  |  |  |  |  |  |
| d170 Writing | 2 (4%) | x |  |  |  |  | x |  |  |  |  |  |  |  |  |  |  |  |
| d310 Communicating with-receiving-spoken messages | 2 (4%) |  | x |  |  |  | x |  |  |  |  |  |  |  |  |  |  |  |
| d360 Using communication devices and techniques | 2 (4%) | x | x |  |  |  |  |  |  |  |  |  |  |  |  |  |  |  |
| d430 Lifting and carrying objects | 2 (4%) | x |  |  | x |  |  |  |  |  |  |  |  |  |  |  |  |  |
| d910 Community life | 2 (4%) |  |  | x | x |  |  |  |  |  |  |  |  |  |  |  |  |  |
| d115 Listening | 1 (2%) |  |  |  |  |  | x |  |  |  |  |  |  |  |  |  |  |  |
| d130 Copying | 1 (2%) | x |  |  |  |  |  |  |  |  |  |  |  |  |  |  |  |  |
| d155 Acquiring skills | 1 (2%) | x |  |  |  |  |  |  |  |  |  |  |  |  |  |  |  |  |
| d330 Speaking | 1 (2%) | x |  |  |  |  |  |  |  |  |  |  |  |  |  |  |  |  |
| d350 Conversation | 1 (2%) |  | x |  |  |  |  |  |  |  |  |  |  |  |  |  |  |  |
| d760 Family relationships | 1 (2%) |  | x |  |  |  |  |  |  |  |  |  |  |  |  |  |  |  |
| d770 Intimate relationships | 1 (2%) |  | x |  |  |  |  |  |  |  |  |  |  |  |  |  |  |  |
| d940 Human rights | 1 (2%) |  |  |  |  |  |  |  |  |  |  |  |  |  |  |  |  | x |
